# Use of the Silastic Sheath in Bladder Neck Reconstruction

**DOI:** 10.1100/tsw.2004.54

**Published:** 2004-06-07

**Authors:** D.A. Diamond, G.F. Quimby, R.C. Rink, P.G. Ransley

**Affiliations:** Harvard Medical School, Boston, MA, USA

**Keywords:** Incontinence, Urinary, Silastic, Bladder Neck, Reconstruction

## Abstract

OBJECTIVE: The study compared two populations of patients undergoing bladder neck reconstruction using the silastic sheath in two major pediatric centers. The success with this technique was markedly different in the two centers. The purpose of the study was to determine factors that might explain the divergent results.PATIENTS AND METHODS: Fifteen patients treated in Indianapolis were compared with 94 patients treated in London with the silastic sheath technique of bladder neck reconstruction. Eighty-seven percent of the Indianapolis patients had myelomeningocele whereas 86% of the London group had exstrophy/epispadias. Median age of the Indianapolis patients was 11 years whereas it was 8.4 years in London. Seventy-three percent of patients in Indianapolis were female and 79% in London were male. Patients were followed for a minimum of eight years in Indianapolis and a mean of seven years in London. Similar surgical technique was employed in the two centers but, over time, the London approach included use of a non-reinforced silastic wrapped loosely around the bladder neck with the interposition of omentum. RESULTS: Both groups achieved continence rates exceeding 90%. Of the Indianapolis patients, two-thirds experienced erosion of the silastic at a mean of 48 months. With modifications in the London technique, the erosion rate of silastic was lowered from 100% to 7%. CONCLUSION: Direct, snug wrap of silastic without omentum around the Young-Dees tube as well as simultaneous bladder augmentation placed patients at increased risk for erosion. The silastic sheath technique may be less applicable to myelomeningocele patients. It seems most applicable to older male patients with exstrophy or epispadias undergoing Young-Dees bladder neck reconstruction who have the ability to void.

## INTRODUCTION

In 1985 Mitchell and Rink proposed a creative modification of bladder neck reconstruction utilizing a Silastic wrap of the Young-Dees tube in order to preserve length, prevent kinking of the tube, and to facilitate subsequent artificial urinary sphincter placement if necessary.[1] Recently, the Indianapolis group presented their long-term experience with this technique, which proved to be discouraging. [2]

Shortly after Mitchellís description, the London group incorporated the Silastic wrap technique in bladder neck reconstruction. [3] This group has also recently analyzed their long-term experience which was more encouraging. [4]

In order to reconcile the differing experiences of these two groups and to arrive at an understanding of the role of Silastic wrap in bladder neck reconstruction, these two series were compared. Attention was focused on differences in patient populations as well as technical differences in the two approaches.

## PATIENTS AND METHODS

The Indianapolis group performed sheath placements in 15 patients, 11 females and 4 males, ranging in age from 2.5 to 27 years with a mean age of 11 years. Thirteen patients (87%) had myelodysplasia, 1 had bladder exstrophy, and 1 epispadias. In 12 cases, sheaths were placed at the time of initial Young-Dees-Leadbetter bladder neck reconstruction, and 3 during procedures subsequent to initial Young-Dees-Leadbetter bladder neck reconstruction (re-do bladder neck, bladder augmentation, removal of an eroded cuff). In 8 cases a simultaneous bladder augmentation was performed at the time of Silastic sheath placement.

The technique of sheath placement entailed use of a .007 inch thickness of reinforced Silastic folded triply into a 1.5cm wide strip. The strip was placed circumferentially directly around the mid-portion of the repair and sutured to itself snugly but not tightly.

The London series included 94 procedures on 84 patients ñ 66 males and 17 females (and 1 penile agenesis patient raised as a female) ranging in age from 15 months to 19 years with a mean age of 8.4 years. Seventy-two patients, or 86%, had classical bladder exstrophy or epispadias. No patients with myelodysplasia were included in the series although two had occult neuropathic bladder. In fifty-one (65%) procedures, the Silastic was placed at initial reconstruction, and in 33 (35%) it represented a repeat reconstruction. Simultaneous bladder augmentation was performed in 41 (49%).

The technique of bladder neck reconstruction originally employed in the London series closely resembled that of the Indianapolis group. A 4cm long sheath of .01 inch reinforced Silastic was wrapped snugly in a single layer directly around the Young-Dees tube. Because of a high erosion rate, the technique underwent a number of modifications. The stiff, reinforced Silastic was abandoned for the more supple non-reinforced Silastic. Initially, a thinner (.005") material was folded in triple thickness to provide bulk, but ultimately, in response to coninuing erosion, non-reinforced silastic in the original thickness (.01") was used in a single layer. The original snug wrap was abandoned for a looser wrap around the bladder neck with the interposition of omentum between the bladder neck and Silastic.

## RESULTS

The Indianapolis series had a 93% short-term continence rate. One patient had an artificial sphincter replaced. Two-thirds of patients experienced erosion of the Silastic into the bladder neck at a mean time of 48 months with only two early erosions at less than six months following sheath placement.

In the London experience, continence rates were quite similar (95%) to those in Indianapolis. Erosion initially occurred in all patients undergoing a snug, wrap of reinforced Silastic directly around the bladder neck. The time interval to erosion in this group was between one and seven months with a mean of 3.4 months. With modifications in the technique to the looser Slastic wrap with omentum, the erosion rate was lowered to 7%. The time interval to erosion in the modified group was 4 to 20 months with a mean of 13.3 months.

The major differences in the two series in terms of patient populations and technical approaches are listed in tables I and II.

**TABLE 1**
**Patient Characteristics**

| | **Indianapolis** | **London** |
|---|---|---|
| **Diagnosis** | (87%) Myelomeningocele | (86%) Exstrophy/Epispadias |
| **Age (mean)** | 11 years | 8.4 years |
| **Sex** | (73%) Female | (79%) Male |
| **Follow-up** | 8 years (minimum) | 7 years (mean) |

**TABLE 2**
**Surgical Technique**

| | **Indianapolis** | **London (Group (III)** |
|---|---|---|
| **Silastic** | Reinforced | Non-reinforced |
| | Triple thickness | Single thickness |
| | 1.5cm long | 2.5cm long |
| **Wrap** | Snug | Loose |
| **Omentum** | No | Yes |
| **Simultaneous Augmentation** | 53% | 66% |

## DISCUSSION

The achievement of urinary continence with bladder neck reconstruction has remained one of the most daunting tasks in pediatric urology. In the most successful series bladder neck reconstruction, alone, has produced continence in 75 to 87% of patients. [(5)] A number of series from major pediatric centers have reported continence rates of 34 to 38% following bladder neck reconstruction, alone, which has been more than doubled to 77 to 80% with the use of adjunctive procedures. [(6-8)] These have included bladder augmentation, use of the artificial urinary sphincter, the Mitrofanoff principle, and clean intermittent catherization.

Given the unreliability of bladder neck reconstruction, Mitchell and Rink in 1985 proposed the concept of wrapping the Young-Dees tube with Silastic. [(1)] Their goals included maintenance of tube length to promote continence, the prevention of tube kinking to allow ease of catheterization, and provision of a safe, surgical plane should artificial urinary sphincter placement be necessary. The early continence rate achieved with this technique was excellent (93%). But erosion of Silastic into the Young-Dees tube occurred at a discouraging rate of 67% over nine years, and has resulted in the abandonment of the approach by its original proponents.

The London experience, over time, was a more encouraging one. The very early results, utilizing an approach almost identical to that originally described by the Indianapolis group resulted in a 100% erosion rate. [(3)] This resulted in several technical modifications made over time, most as a result of trial and error, ultimately lowering the erosion rate to its current 7%.[(4)]

In assessing potential risk factors for Silastic erosion into the Young-Dees tube at least three mechanisms seemed plausible ñ ischemic necrosis, infection, and iatrogenic injury.

The 100% erosion rate in the early London experience was likely the result of ischemic necrosis. In these patients, significant dissection posterior to the Young-Dees tube was required to placea 4cm long, stiff reinforced Silastic sheath. With erosion occurring at a mean of 3.7 months in all of these patients, an incentive for immediate modification in the technique was provided. Thus, the London group moved to a looser wrap with the interposition of omentum between bladder neck and Silastic. In the Indianapolis experience erosion tended to be diagnosed later, but it is likely that ischemic necrosis was an important factor. It is possible that erosion had occurred much earlier than it was detected as the predominant presentation in this group was recurrent urinary tract infection rather than difficulty with intermittent catheterization or urinary incontinence. With the snug Silastic wrap dirctly around the bladder neck, increased girth of the Young-Dees tube may have been poorly accommodated. Somatic growth or increased bladder wall thickness and lack of sensation with intermittent catheterization in the predominantly myelomeningocele population may have predisposed to ischemic necrosis. The higher mean age (11 years) in the Indianapolis series may have been consistent with a pubertal growth spurt in some patients, potentially accelerating these processes. Because of the delay in sheath erosion in the Indianapolis experience, there was not the early incentive to modify surgical technique.

Over time, London moved away from the stiffer, reinforced Silastic to the more supple, non-reinforced material in a single layer. The original, snug wrap evolved into a loose wrap. These two factors would seem to have lessened the risk of ischemic necrosis and in particular to have allowed for growth an increased tube girth.

The interposition of omentum may have contributed to reducing erosion in a number of ways. An additional, pliable tissue layer separated the Silastic from the Young-Dees tube. Given its rich lymphatic supply, omentum may have contributed to absorption of residual hematoma or urine extravasation and thus reduced the risk of infection. Furthermore, omentum encasing both bladder neck and Silastic may have prevented rigid scar formation. At cystoscopy, Young-Dees tubes wrapped with omentum seemed particularly pliable. In this respect, one of the original goals ñ to prevent kinking of the Young-Dees tube ñ was likely achieved. Young-Dees tube length was not noticeably enhanced, however.

Infection is also likely to have been an important risk factor for erosion. In the analysis of their own series, the London group found simultaneous bladder augmentation to be a statistically significant risk factor, in the patients with triply folded silastic. These erosions tended to occur early. Seventy-five percent of the Indianapolis patients undergoing simultaneous bladder augmentation suffered erosion, although the timing of erosion was unclear. We hypothesize that infection of the Silastic sheath was a contributing factor to erosion in these patients.

The one well documented iatrogenic cause was erosion following internal urethrotomy in the London series. In the two series, the majority of patients were on clean intermittent catherization, which may also have contributed to erosion in certain patients. This may have been particularly true in the Indianapolis group in which injury from catheterization in those with lack of sensation was not uncommon.

Based on these two series, it would seem that the direct wrap of Silastic (without omentum) around the Young-Dees tube, particularly when wrapped snugly, as well as simultaneous bladder augmentation with Silastic sheath placement put patients at increased risk for erosion. The Silastic sheath technique may be less applicable to myelomeningocele patients, although the modified approach using omentum and a loose wrap of single thickness, non-reinforced Silastic remains untested in this group and theoretically attractive. While this technique has not, in itself, resulted in increased continence rates, to date this approach has proved most valuable to 16% of patients in the London series who have undergone artificial urinary sphincter placement. As a group, these have consisted of older male patients with exstrophy or epispadias undergoing Young-Dees bladder neck reconstruction who have the ability to void. While approximately half of these patients have been augmented, the Silastic sheath technique would seem best suited to the unaugmented population with a reduced risk of infection and a greater likelihood of spontaneous voiding.
